# 2-(3,4-Dimethyl-5,5-dioxo-2*H*,4*H*-pyrazolo­[4,3-*c*][1,2]benzothia­zin-2-yl)-*N*-(3-meth­oxy­benz­yl)acetamide

**DOI:** 10.1107/S1600536812040226

**Published:** 2012-10-03

**Authors:** Matloob Ahmad, Hamid Latif Siddiqui, Muhammad Zia-ur-Rehman, Sana Aslam, Masood Parvez

**Affiliations:** a Department of Chemistry, Government College University, Faisalabad 38000, Pakistan; b Institute of Chemistry, University of the Punjab, Lahore 54590, Pakistan; c Applied Chemistry Research Center, PCSIR Laboratories Complex, Lahore 54600, Pakistan; d Department of Chemistry, The University of Calgary, 2500 University Drive NW, Calgary, Alberta, Canada T2N 1N4

## Abstract

The asymmetric unit of the title compound, C_21_H_22_N_4_O_4_S, contains two mol­ecules (*A* and *B*), in which the thia­zine rings adopt an *S*-envelope conformation with the S atoms displaced by 0.621 (2) and 0.697 (2) Å from the mean planes formed by the remaining ring atoms. The dihedral angles between the *N*-methyl­acetamide groups and the meth­oxy­benzene rings are 8.67 (10) and 54.49 (6)° in the two mol­ecules and the equivalent torsion angles in the *N*-methyl­acetamide chains connecting the ring systems also differ. In the crystal, N—H⋯O hydrogen bonds connect the components into *C*(4) [100] chains of alternating *A* and *B* mol­ecules. The packing is consolidated by weak C—H⋯O inter­actions, which generate a three-dimensional network.

## Related literature

For therapeutic applications of benzothia­zines, see: Turck *et al.* (1996) ▶; Lombardino *et al.* (1973 ▶); Zinnes *et al.* (1973 ▶). For therapeutic applications of pyrrazoles, see: Silverstein *et al.* (2000 ▶). For the properties and crystal structures of related pyrazolo­benzothia­zine derivatives, see: Ahmad *et al.* (2010*a* ▶,*b* ▶, 2012 ▶; 2011*a* ▶,*b* ▶).
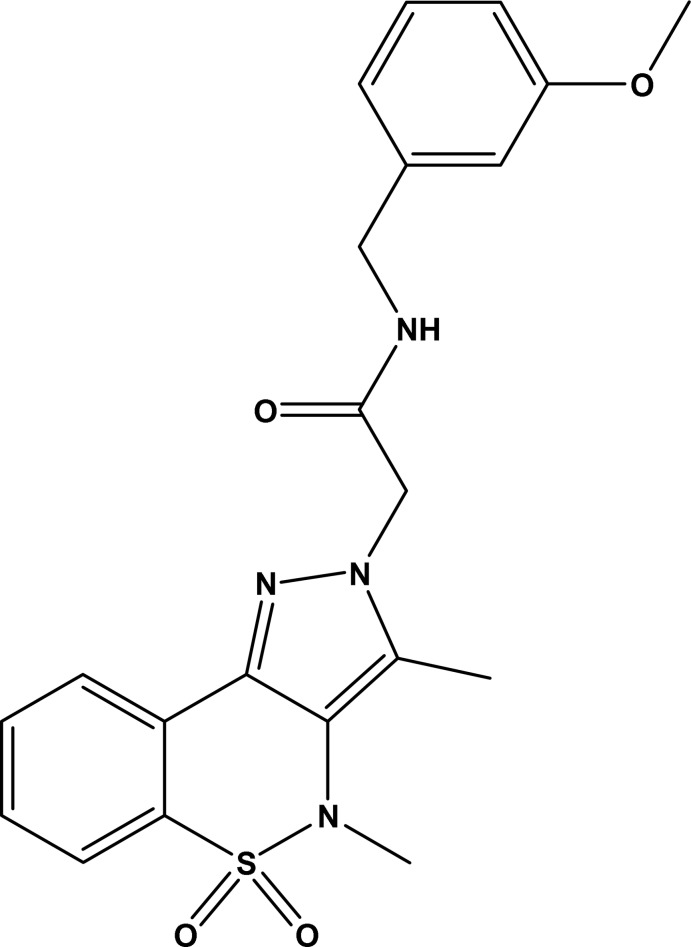


## Experimental

### 

#### Crystal data

C_21_H_22_N_4_O_4_S
*M* *_r_* = 426.49
Monoclinic, 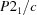

*a* = 8.6541 (1) Å
*b* = 25.8809 (3) Å
*c* = 18.3892 (2) Å
β = 92.208 (1)°
*V* = 4115.68 (8) Å^3^
*Z* = 8
Cu *K*α radiation
μ = 1.71 mm^−1^
*T* = 173 K
0.14 × 0.12 × 0.02 mm

#### Data collection

Bruker APEXII CCD diffractometer
Absorption correction: multi-scan (*SADABS*; Bruker, 2004 ▶) *T*_min_ = 0.796, *T*_max_ = 0.967
70993 measured reflections
7975 independent reflections
6621 reflections with *I* > 2σ(*I*)
*R*_int_ = 0.049

#### Refinement

*R*[*F* ^2^ > 2σ(*F* ^2^)] = 0.036
*wR*(*F* ^2^) = 0.098
*S* = 1.02
7975 reflections
547 parameters
H-atom parameters constrained
Δρ_max_ = 0.31 e Å^−3^
Δρ_min_ = −0.37 e Å^−3^

### 

Data collection: *APEX2* (Bruker, 2004 ▶); cell refinement: *SAINT* (Bruker, 2004 ▶); data reduction: *SAINT*; program(s) used to solve structure: *SHELXS97* (Sheldrick, 2008 ▶); program(s) used to refine structure: *SHELXL97* (Sheldrick, 2008 ▶); molecular graphics: *ORTEP-3 for Windows* (Farrugia, 1997 ▶); software used to prepare material for publication: *SHELXL97*.

## Supplementary Material

Crystal structure: contains datablock(s) global, I. DOI: 10.1107/S1600536812040226/hb6961sup1.cif

Structure factors: contains datablock(s) I. DOI: 10.1107/S1600536812040226/hb6961Isup2.hkl

Supplementary material file. DOI: 10.1107/S1600536812040226/hb6961Isup3.cml

Additional supplementary materials:  crystallographic information; 3D view; checkCIF report

## Figures and Tables

**Table 1:** Hydrogen-bond geometry (Å, °)

*D*—H⋯*A*	*D*—H	H⋯*A*	*D*⋯*A*	*D*—H⋯*A*
N4—H4*N*⋯O7^i^	0.88	2.12	2.9511 (17)	158
N8—H8*N*⋯O3^ii^	0.88	2.02	2.8928 (18)	171
C10—H10*C*⋯O6^iii^	0.98	2.45	3.349 (2)	152
C12—H12*A*⋯O7^i^	0.99	2.56	3.2099 (19)	123
C14—H14*A*⋯N6^ii^	0.99	2.58	3.556 (2)	168
C16—H16⋯O7^i^	0.95	2.43	3.369 (2)	172
C25—H25⋯O4^iv^	0.95	2.47	3.365 (2)	156
C31—H31*C*⋯O2^v^	0.98	2.49	3.203 (2)	129
C33—H33*A*⋯O3^ii^	0.99	2.46	3.320 (2)	145
C33—H33*B*⋯O5^vi^	0.99	2.45	3.352 (2)	151
C40—H40⋯O2	0.95	2.59	3.367 (3)	139

Symmetry codes: (i) 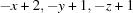; (ii) 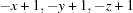; (iii) 
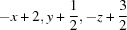
; (iv) 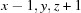; (v) 
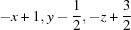
; (vi) 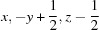.

**Fig. 1.**
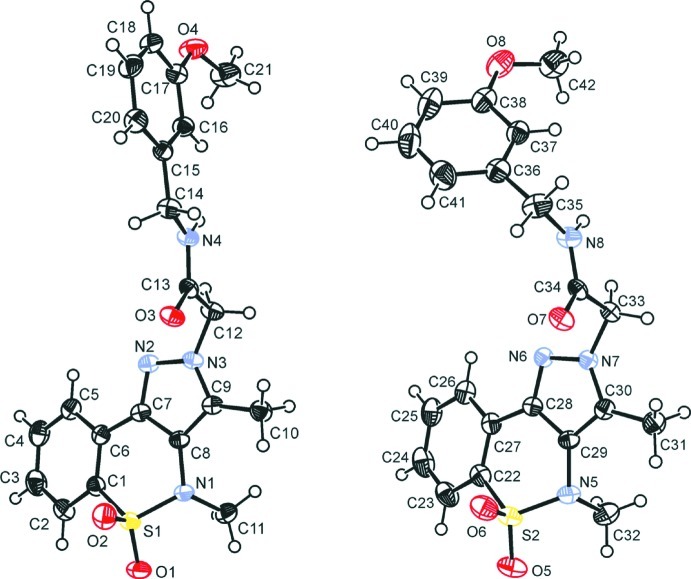
The asymmetric unit of the title compound with displacement ellipsoids drawn at the 50% probability level. H atoms are presented as small spheres of arbitrary radius.

**Fig. 2.**
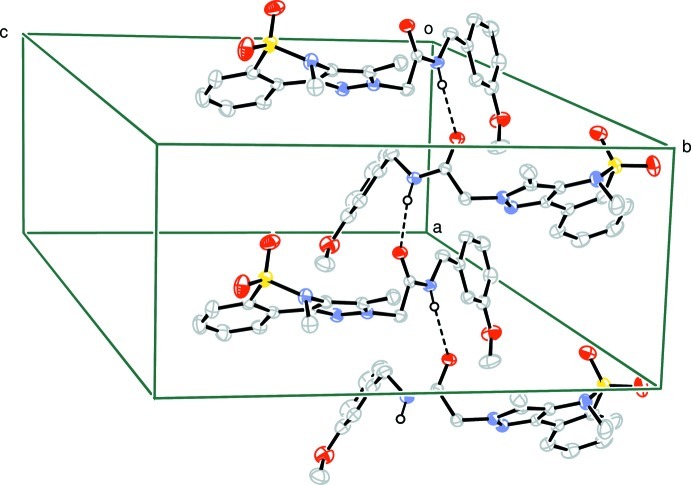
Part of the crystal structure with N—H···O hydrogen bonds shown as dashed lines. H atoms non-participating in hydrogen-bonding are omitted for clarity.

**Fig. 3.**
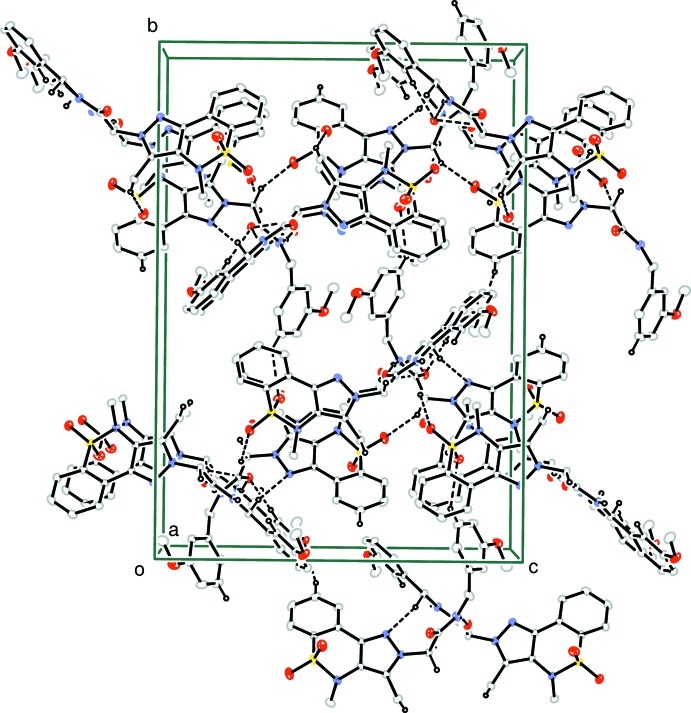
A view of the hydrogen bonding interactions (dotted lines) in the crystal structure of the title compound. H atoms non-participating in hydrogen-bonding were omitted for clarity.

## supplementary crystallographic information

### Comment

1,2-Benzothiazines are the core nuclei for their potent anti-inflammatory and analgesic drugs, known as oxicams (Turck *et al.*, 1996; Lombardino *et al.*, 1973; Zinnes *et al.*, 1973). On the other hand, celecoxib, a pyrazole compound is an anti-inflammatory drug and a selective inhibitor of cox-2 enzyme (Silverstein *et al.*, 2000). These structural and bioactivity features have led us to the synthesis of new carboxamides based on pyrazolobenzothiazines, which are structural hybrids of both of these medicinally important heterocycles (Ahmad *et al.*, 2010*a*; 2010*b*, 2012). We report the synthesis and crystal structure of the title compound in this article.

The asymmetric unit of the title compound contains two independent conformers (Fig. 1). In both molecules, the heterocyclic thiazine rings adopt S-envelope conformations with atoms S1 and S2 displaced by 0.621 (2) and 0.697 (2) Å, respectively, from the mean planes formed by the remaining ring atoms (rmsd 0.0469 and 0.0608 Å, respectively). In both molecules, the atoms N1–N3/C1/C6–C9 and N5–N6/C22/C27–C30 of the thiazine and pyrrazole rings are individually close to coplanar (rmsd 0.0392 and 0.0548 Å) and form dihedral angles 14.01 (7) and 15.69 (8)° with the mean-planes of the benzene rings (C1–C6) and (C22–C27), respectively. The *N*-methylacetamide groups (O3/N4/C12–C14) and (O7/N8/C33–C35)) are individually almost planar (rmsd 0.0229 and 0.0047 Å, respectively) and their mean-planes are oriented at drastically different angles, 8.67 (10) and 54.49 (6)°, with respect to the benzene rings (C15–C20) and (C36–C41), respectively. The conformational differences in the two molecules are more pronounced from a comparison of torsion angles involving the *N*-methylacetamide chain connecting the methoxy benzene and pyrrazole rings, *e.g.*, in one molecule the torsion angles, N2/N3–C12/C13, N3/C12–C13/N4, C12/C13–N4/C14, C13/N4–C14/C15 and N4/C14–C15/C16 are: 82.23 (17), -154.08 (14), -175.58 (14), -171.18 (14) and -13.3 (2)°, respectively. The corresponnding torsion angles in the second molecule are: 72.54 (17), -136.44 (14), 179.52 (15), -132.81 (17) and -61.8 (2)°, respectively. The bond distances and angles in both molecules of the title compound agree very well with the corresponding bond distances and angles reported in closely related compounds (Ahmad *et al.*, 2010*b*; Ahmad *et al.*, 2011*a*; Ahmad *et al.*, 2011*b*).

The crystal structure features intermolecular hydrogen bonding interactions N4—H4N···O7 and N8—H8N···O3 resulting in chains of molecules extended along [100] (Fig. 2 & Tab. 1). The crystal structure is further consolidated by weak C—H···O hydrogen bonds, which result in a 3-D network (Fig. 3 & Tab. 1).

#——————————————————————————

### Experimental

3,4-Dimethyl-5,5-dioxidopyrazolo[4,3-*c*][1,2] benzothiazin-2(4*H*)-yl acetic acid (1.013 g, 3.3 mmoles) was dissolved in toluene:THF (2:1) and boran–THF complex (1.1 mmoles) was added ton it. The reaction mixture was stirred for a period of 40 minutes and 3-methoxybenzyl amine (0.45 g, 3.3 mmoles) was added to it. The contents of the flask were refluxed for 5 hrs. The solvent was evaporated under vacuum and the title compound as the end product was purified by column chromatography. Colourless plates were grown from ethyl acetate solution at room temperature by slow evaporation; m.p. 425–426 K.

### Refinement

All H atoms were positioned geometrically and refined using a riding model, with N—H = 0.88 Å and C—H = 0.95, 0.98 and 0.99 Å, for aryl, methyl and methylene type H-atoms, respectively. The *U*_iso_(H) were allowed at 1.2*U*_eq_(N/C).

### Crystal data

**Table:**

C_21_H_22_N_4_O_4_S	*F*(000) = 1792
*M**_r_* = 426.49	*D*_x_ = 1.377 Mg m^−^^3^
Monoclinic, *P*2_1_/*c*	Cu *K*α radiation, λ = 1.54178 Å
Hall symbol: -P 2ybc	Cell parameters from 9915 reflections
*a* = 8.6541 (1) Å	θ = 3.4–71.7°
*b* = 25.8809 (3) Å	µ = 1.71 mm^−^^1^
*c* = 18.3892 (2) Å	*T* = 173 K
β = 92.208 (1)°	Plate, colorless
*V* = 4115.68 (8) Å^3^	0.14 × 0.12 × 0.02 mm
*Z* = 8	

### Data collection

**Table:**

Bruker APEXII CCD diffractometer	7975 independent reflections
Radiation source: fine-focus sealed tube	6621 reflections with *I* > 2σ(*I*)
Graphite monochromator	*R*_int_ = 0.049
ω and φ scans	θ_max_ = 72.7°, θ_min_ = 3.0°
Absorption correction: multi-scan (*SADABS*; Bruker, 2004)	*h* = −10→10
*T*_min_ = 0.796, *T*_max_ = 0.967	*k* = −31→32
70993 measured reflections	*l* = −22→22

### Refinement

**Table:**

Refinement on *F*^2^	Primary atom site location: structure-invariant direct methods
Least-squares matrix: full	Secondary atom site location: difference Fourier map
*R*[*F*^2^ > 2σ(*F*^2^)] = 0.036	Hydrogen site location: inferred from neighbouring sites
*wR*(*F*^2^) = 0.098	H-atom parameters constrained
*S* = 1.02	*w* = 1/[σ^2^(*F*_o_^2^) + (0.0486*P*)^2^ + 1.780*P*] where *P* = (*F*_o_^2^ + 2*F*_c_^2^)/3
7975 reflections	(Δ/σ)_max_ = 0.001
547 parameters	Δρ_max_ = 0.31 e Å^−^^3^
0 restraints	Δρ_min_ = −0.37 e Å^−^^3^

### Special details

**Table:**

Geometry. All e.s.d.'s (except the e.s.d. in the dihedral angle between two l.s. planes) are estimated using the full covariance matrix. The cell e.s.d.'s are taken into account individually in the estimation of e.s.d.'s in distances, angles and torsion angles; correlations between e.s.d.'s in cell parameters are only used when they are defined by crystal symmetry. An approximate (isotropic) treatment of cell e.s.d.'s is used for estimating e.s.d.'s involving l.s. planes.
Refinement. Refinement of *F*^2^ against ALL reflections. The weighted *R*-factor *wR* and goodness of fit *S* are based on *F*^2^, conventional *R*-factors *R* are based on *F*, with *F* set to zero for negative *F*^2^. The threshold expression of *F*^2^ > σ(*F*^2^) is used only for calculating *R*-factors(gt) *etc*. and is not relevant to the choice of reflections for refinement. *R*-factors based on *F*^2^ are statistically about twice as large as those based on *F*, and *R*- factors based on ALL data will be even larger.

### Fractional atomic coordinates and isotropic or equivalent isotropic displacement parameters (Å^2^)

**Table:**

	*x*	*y*	*z*	*U*_iso_*/*U*_eq_	
S1	0.73006 (5)	0.718057 (16)	0.68662 (2)	0.03077 (11)	
O1	0.73608 (18)	0.75309 (5)	0.74693 (7)	0.0448 (3)	
O2	0.58770 (14)	0.69282 (5)	0.66768 (8)	0.0427 (3)	
O3	0.66878 (13)	0.64371 (5)	0.36992 (6)	0.0305 (3)	
O4	1.13937 (15)	0.51550 (5)	0.09408 (7)	0.0442 (3)	
N1	0.78516 (16)	0.74988 (5)	0.61494 (7)	0.0283 (3)	
N2	0.94469 (15)	0.64777 (5)	0.50587 (7)	0.0268 (3)	
N3	0.90464 (15)	0.68467 (5)	0.45640 (7)	0.0258 (3)	
N4	0.83869 (15)	0.61454 (5)	0.28889 (7)	0.0257 (3)	
H4N	0.9357	0.6140	0.2762	0.031*	
C1	0.86939 (19)	0.66886 (6)	0.70074 (9)	0.0277 (3)	
C2	0.9089 (2)	0.65336 (7)	0.77123 (10)	0.0379 (4)	
H2	0.8681	0.6709	0.8116	0.045*	
C3	1.0082 (2)	0.61207 (8)	0.78196 (11)	0.0439 (5)	
H3	1.0366	0.6012	0.8300	0.053*	
C4	1.0666 (2)	0.58638 (7)	0.72289 (11)	0.0398 (4)	
H4	1.1326	0.5574	0.7308	0.048*	
C5	1.02988 (19)	0.60238 (6)	0.65256 (10)	0.0310 (4)	
H5	1.0717	0.5847	0.6126	0.037*	
C6	0.93145 (18)	0.64456 (6)	0.64015 (9)	0.0252 (3)	
C7	0.90162 (17)	0.66776 (6)	0.56918 (8)	0.0238 (3)	
C8	0.83336 (18)	0.71665 (6)	0.55833 (8)	0.0242 (3)	
C9	0.83390 (18)	0.72636 (6)	0.48488 (8)	0.0247 (3)	
C10	0.7748 (2)	0.77070 (7)	0.44095 (9)	0.0324 (4)	
H10A	0.7004	0.7902	0.4689	0.039*	
H10B	0.7238	0.7579	0.3960	0.039*	
H10C	0.8612	0.7932	0.4289	0.039*	
C11	0.8664 (2)	0.79951 (7)	0.62507 (10)	0.0343 (4)	
H11A	0.8964	0.8127	0.5777	0.041*	
H11B	0.9590	0.7944	0.6566	0.041*	
H11C	0.7977	0.8244	0.6477	0.041*	
C12	0.93011 (19)	0.67463 (7)	0.38043 (8)	0.0288 (3)	
H12A	1.0292	0.6560	0.3760	0.035*	
H12B	0.9377	0.7078	0.3541	0.035*	
C13	0.79951 (18)	0.64258 (6)	0.34597 (8)	0.0237 (3)	
C14	0.72376 (19)	0.58481 (7)	0.24711 (9)	0.0303 (4)	
H14A	0.6516	0.6091	0.2218	0.036*	
H14B	0.6632	0.5642	0.2813	0.036*	
C15	0.78873 (19)	0.54888 (6)	0.19144 (8)	0.0260 (3)	
C16	0.94083 (19)	0.55117 (6)	0.16971 (9)	0.0290 (3)	
H16	1.0103	0.5759	0.1909	0.035*	
C17	0.9909 (2)	0.51720 (7)	0.11689 (9)	0.0315 (4)	
C18	0.8897 (2)	0.48220 (7)	0.08388 (9)	0.0350 (4)	
H18	0.9240	0.4596	0.0471	0.042*	
C19	0.7390 (2)	0.48049 (7)	0.10484 (10)	0.0381 (4)	
H19	0.6688	0.4567	0.0821	0.046*	
C20	0.6881 (2)	0.51308 (7)	0.15891 (10)	0.0339 (4)	
H20	0.5844	0.5109	0.1737	0.041*	
C21	1.2412 (2)	0.55582 (9)	0.11614 (13)	0.0518 (5)	
H21A	1.3392	0.5519	0.0917	0.062*	
H21B	1.2605	0.5542	0.1690	0.062*	
H21C	1.1945	0.5892	0.1030	0.062*	
S2	0.75867 (5)	0.300172 (18)	1.06466 (2)	0.03455 (11)	
O5	0.77325 (18)	0.27220 (6)	1.13161 (7)	0.0496 (4)	
O6	0.88416 (15)	0.33157 (6)	1.04272 (7)	0.0425 (3)	
O7	0.84261 (13)	0.35681 (5)	0.74617 (6)	0.0311 (3)	
O8	0.25890 (16)	0.51780 (5)	0.54673 (8)	0.0489 (4)	
N5	0.72180 (17)	0.25814 (6)	0.99927 (7)	0.0318 (3)	
N6	0.54399 (15)	0.34265 (5)	0.86314 (7)	0.0248 (3)	
N7	0.61082 (15)	0.30530 (5)	0.82299 (7)	0.0240 (3)	
N8	0.64844 (16)	0.38308 (6)	0.66855 (8)	0.0308 (3)	
H8N	0.5534	0.3765	0.6524	0.037*	
C22	0.5931 (2)	0.34022 (7)	1.06449 (9)	0.0307 (4)	
C23	0.5371 (2)	0.35857 (8)	1.12924 (10)	0.0404 (4)	
H23	0.5812	0.3473	1.1746	0.048*	
C24	0.4164 (2)	0.39335 (8)	1.12678 (11)	0.0457 (5)	
H24	0.3766	0.4060	1.1708	0.055*	
C25	0.3525 (2)	0.41003 (7)	1.06055 (11)	0.0411 (4)	
H25	0.2717	0.4349	1.0595	0.049*	
C26	0.4056 (2)	0.39074 (7)	0.99589 (10)	0.0328 (4)	
H26	0.3599	0.4020	0.9508	0.039*	
C27	0.52598 (19)	0.35489 (6)	0.99667 (9)	0.0273 (3)	
C28	0.57863 (18)	0.32881 (6)	0.93188 (8)	0.0240 (3)	
C29	0.66903 (18)	0.28375 (6)	0.93412 (9)	0.0256 (3)	
C30	0.68912 (18)	0.26942 (6)	0.86324 (9)	0.0257 (3)	
C31	0.7701 (2)	0.22448 (7)	0.83220 (10)	0.0354 (4)	
H31A	0.8316	0.2071	0.8708	0.042*	
H31B	0.8385	0.2364	0.7944	0.042*	
H31C	0.6939	0.2003	0.8109	0.042*	
C32	0.6376 (3)	0.21044 (7)	1.01727 (11)	0.0452 (5)	
H32A	0.6335	0.1874	0.9749	0.054*	
H32B	0.5322	0.2192	1.0305	0.054*	
H32C	0.6912	0.1930	1.0583	0.054*	
C33	0.60293 (19)	0.30988 (6)	0.74456 (8)	0.0257 (3)	
H33A	0.4954	0.3177	0.7278	0.031*	
H33B	0.6333	0.2767	0.7225	0.031*	
C34	0.71003 (17)	0.35267 (6)	0.72004 (8)	0.0236 (3)	
C35	0.7295 (2)	0.42690 (8)	0.63715 (11)	0.0396 (4)	
H35A	0.7603	0.4179	0.5874	0.048*	
H35B	0.8246	0.4343	0.6670	0.048*	
C36	0.6281 (2)	0.47419 (7)	0.63410 (10)	0.0354 (4)	
C37	0.4907 (2)	0.47291 (7)	0.59220 (9)	0.0326 (4)	
H37	0.4611	0.4421	0.5672	0.039*	
C38	0.3965 (2)	0.51621 (7)	0.58663 (10)	0.0387 (4)	
C39	0.4419 (3)	0.56167 (8)	0.62194 (13)	0.0509 (5)	
H39	0.3795	0.5918	0.6172	0.061*	
C40	0.5767 (3)	0.56283 (8)	0.66352 (13)	0.0565 (6)	
H40	0.6066	0.5938	0.6879	0.068*	
C41	0.6705 (3)	0.51915 (9)	0.67061 (12)	0.0496 (5)	
H41	0.7628	0.5202	0.7002	0.059*	
C42	0.2039 (2)	0.47010 (9)	0.51694 (13)	0.0520 (5)	
H42A	0.1065	0.4760	0.4893	0.062*	
H42B	0.1867	0.4456	0.5565	0.062*	
H42C	0.2807	0.4559	0.4846	0.062*	

### Atomic displacement parameters (Å^2^)

**Table:**

	*U*^11^	*U*^22^	*U*^33^	*U*^12^	*U*^13^	*U*^23^
S1	0.0327 (2)	0.0311 (2)	0.0291 (2)	0.00564 (16)	0.00925 (17)	−0.00061 (15)
O1	0.0658 (9)	0.0403 (7)	0.0290 (6)	0.0125 (7)	0.0132 (6)	−0.0043 (5)
O2	0.0280 (6)	0.0434 (8)	0.0573 (8)	0.0044 (6)	0.0111 (6)	0.0064 (6)
O3	0.0220 (6)	0.0431 (7)	0.0266 (6)	−0.0017 (5)	0.0033 (5)	−0.0041 (5)
O4	0.0383 (7)	0.0459 (8)	0.0493 (8)	−0.0035 (6)	0.0146 (6)	−0.0187 (6)
N1	0.0340 (8)	0.0260 (7)	0.0250 (7)	0.0039 (6)	0.0045 (6)	−0.0037 (5)
N2	0.0250 (7)	0.0284 (7)	0.0267 (7)	0.0026 (5)	−0.0011 (6)	−0.0039 (5)
N3	0.0229 (7)	0.0314 (7)	0.0229 (6)	0.0013 (5)	0.0001 (5)	−0.0040 (5)
N4	0.0201 (6)	0.0330 (7)	0.0242 (6)	−0.0010 (5)	0.0029 (5)	−0.0037 (5)
C1	0.0274 (8)	0.0274 (8)	0.0285 (8)	−0.0017 (6)	0.0026 (7)	0.0018 (6)
C2	0.0462 (11)	0.0396 (10)	0.0281 (9)	−0.0011 (8)	0.0040 (8)	0.0022 (7)
C3	0.0519 (12)	0.0451 (11)	0.0342 (10)	0.0007 (9)	−0.0046 (9)	0.0124 (8)
C4	0.0384 (10)	0.0344 (10)	0.0461 (11)	0.0049 (8)	−0.0058 (9)	0.0098 (8)
C5	0.0269 (8)	0.0289 (8)	0.0372 (9)	0.0005 (7)	0.0003 (7)	−0.0003 (7)
C6	0.0217 (7)	0.0251 (8)	0.0287 (8)	−0.0034 (6)	−0.0013 (6)	−0.0008 (6)
C7	0.0192 (7)	0.0260 (8)	0.0262 (8)	0.0000 (6)	−0.0009 (6)	−0.0041 (6)
C8	0.0220 (7)	0.0263 (8)	0.0243 (7)	0.0021 (6)	0.0001 (6)	−0.0032 (6)
C9	0.0199 (7)	0.0285 (8)	0.0256 (8)	0.0005 (6)	−0.0005 (6)	−0.0037 (6)
C10	0.0324 (9)	0.0363 (9)	0.0286 (8)	0.0050 (7)	0.0009 (7)	0.0023 (7)
C11	0.0415 (10)	0.0282 (8)	0.0332 (9)	−0.0004 (7)	0.0007 (8)	−0.0053 (7)
C12	0.0243 (8)	0.0378 (9)	0.0244 (8)	−0.0029 (7)	0.0034 (7)	−0.0061 (7)
C13	0.0214 (7)	0.0286 (8)	0.0209 (7)	0.0018 (6)	0.0001 (6)	0.0031 (6)
C14	0.0234 (8)	0.0382 (9)	0.0292 (8)	−0.0033 (7)	0.0004 (7)	−0.0069 (7)
C15	0.0283 (8)	0.0270 (8)	0.0225 (7)	0.0001 (6)	−0.0016 (7)	0.0009 (6)
C16	0.0288 (8)	0.0305 (8)	0.0277 (8)	−0.0035 (7)	0.0003 (7)	−0.0043 (6)
C17	0.0340 (9)	0.0322 (9)	0.0286 (8)	−0.0001 (7)	0.0048 (7)	−0.0016 (7)
C18	0.0464 (11)	0.0294 (9)	0.0293 (9)	−0.0002 (8)	0.0014 (8)	−0.0062 (7)
C19	0.0430 (10)	0.0327 (9)	0.0381 (10)	−0.0089 (8)	−0.0053 (8)	−0.0071 (7)
C20	0.0290 (9)	0.0364 (9)	0.0361 (9)	−0.0053 (7)	−0.0015 (8)	−0.0017 (7)
C21	0.0338 (10)	0.0597 (13)	0.0632 (14)	−0.0082 (9)	0.0192 (10)	−0.0177 (11)
S2	0.0328 (2)	0.0449 (3)	0.0257 (2)	−0.00011 (18)	−0.00307 (17)	0.00447 (17)
O5	0.0581 (9)	0.0620 (9)	0.0283 (7)	0.0026 (7)	−0.0064 (6)	0.0098 (6)
O6	0.0301 (7)	0.0562 (8)	0.0409 (7)	−0.0053 (6)	−0.0041 (6)	0.0006 (6)
O7	0.0211 (6)	0.0437 (7)	0.0284 (6)	−0.0027 (5)	0.0003 (5)	−0.0008 (5)
O8	0.0414 (8)	0.0413 (8)	0.0641 (9)	0.0124 (6)	0.0046 (7)	0.0058 (7)
N5	0.0333 (8)	0.0337 (8)	0.0283 (7)	0.0045 (6)	−0.0008 (6)	0.0066 (6)
N6	0.0235 (7)	0.0260 (7)	0.0252 (7)	0.0017 (5)	0.0032 (5)	−0.0012 (5)
N7	0.0223 (6)	0.0269 (7)	0.0230 (6)	0.0019 (5)	0.0027 (5)	−0.0013 (5)
N8	0.0217 (7)	0.0377 (8)	0.0327 (7)	−0.0052 (6)	−0.0018 (6)	0.0060 (6)
C22	0.0307 (9)	0.0338 (9)	0.0278 (8)	−0.0063 (7)	0.0047 (7)	−0.0002 (7)
C23	0.0463 (11)	0.0487 (11)	0.0267 (9)	−0.0112 (9)	0.0074 (8)	−0.0039 (8)
C24	0.0496 (12)	0.0515 (12)	0.0374 (10)	−0.0103 (10)	0.0204 (9)	−0.0148 (9)
C25	0.0376 (10)	0.0367 (10)	0.0501 (11)	−0.0014 (8)	0.0144 (9)	−0.0108 (8)
C26	0.0308 (9)	0.0309 (9)	0.0371 (9)	−0.0012 (7)	0.0066 (8)	−0.0020 (7)
C27	0.0267 (8)	0.0278 (8)	0.0278 (8)	−0.0052 (6)	0.0069 (7)	0.0001 (6)
C28	0.0213 (7)	0.0270 (8)	0.0239 (8)	−0.0017 (6)	0.0029 (6)	0.0018 (6)
C29	0.0225 (8)	0.0282 (8)	0.0262 (8)	0.0019 (6)	0.0006 (6)	0.0037 (6)
C30	0.0192 (7)	0.0272 (8)	0.0307 (8)	0.0012 (6)	0.0011 (6)	−0.0001 (6)
C31	0.0305 (9)	0.0364 (9)	0.0392 (9)	0.0102 (7)	−0.0003 (8)	−0.0047 (7)
C32	0.0568 (13)	0.0354 (10)	0.0434 (11)	−0.0003 (9)	0.0001 (10)	0.0115 (8)
C33	0.0247 (8)	0.0304 (8)	0.0219 (7)	−0.0012 (6)	−0.0003 (6)	−0.0024 (6)
C34	0.0206 (7)	0.0314 (8)	0.0191 (7)	0.0004 (6)	0.0036 (6)	−0.0055 (6)
C35	0.0276 (9)	0.0452 (11)	0.0460 (11)	−0.0052 (8)	0.0027 (8)	0.0148 (8)
C36	0.0342 (9)	0.0364 (9)	0.0361 (9)	−0.0084 (7)	0.0065 (8)	0.0056 (7)
C37	0.0343 (9)	0.0298 (9)	0.0343 (9)	−0.0024 (7)	0.0076 (8)	0.0021 (7)
C38	0.0377 (10)	0.0360 (10)	0.0431 (10)	0.0017 (8)	0.0120 (8)	0.0041 (8)
C39	0.0550 (13)	0.0318 (10)	0.0672 (14)	0.0017 (9)	0.0199 (11)	−0.0030 (9)
C40	0.0708 (16)	0.0388 (11)	0.0610 (14)	−0.0169 (11)	0.0181 (12)	−0.0129 (10)
C41	0.0510 (12)	0.0506 (12)	0.0471 (11)	−0.0193 (10)	0.0016 (10)	−0.0020 (9)
C42	0.0365 (11)	0.0541 (13)	0.0649 (14)	0.0035 (9)	−0.0064 (10)	0.0050 (11)

### Geometric parameters (Å, º)

**Table:**

S1—O2	1.4257 (14)	S2—O6	1.4270 (14)
S1—O1	1.4319 (13)	S2—O5	1.4296 (13)
S1—N1	1.6406 (14)	S2—N5	1.6436 (15)
S1—C1	1.7659 (17)	S2—C22	1.7684 (18)
O3—C13	1.2302 (19)	O7—C34	1.2314 (19)
O4—C17	1.367 (2)	O8—C38	1.375 (3)
O4—C21	1.415 (2)	O8—C42	1.425 (3)
N1—C8	1.4250 (19)	N5—C29	1.429 (2)
N1—C11	1.473 (2)	N5—C32	1.478 (2)
N2—C7	1.340 (2)	N6—C28	1.337 (2)
N2—N3	1.3547 (19)	N6—N7	1.3592 (18)
N3—C9	1.356 (2)	N7—C30	1.353 (2)
N3—C12	1.4462 (19)	N7—C33	1.4461 (19)
N4—C13	1.331 (2)	N8—C34	1.327 (2)
N4—C14	1.453 (2)	N8—C35	1.464 (2)
N4—H4N	0.8800	N8—H8N	0.8800
C1—C2	1.387 (2)	C22—C23	1.387 (2)
C1—C6	1.404 (2)	C22—C27	1.408 (2)
C2—C3	1.381 (3)	C23—C24	1.378 (3)
C2—H2	0.9500	C23—H23	0.9500
C3—C4	1.385 (3)	C24—C25	1.387 (3)
C3—H3	0.9500	C24—H24	0.9500
C4—C5	1.383 (3)	C25—C26	1.384 (2)
C4—H4	0.9500	C25—H25	0.9500
C5—C6	1.398 (2)	C26—C27	1.395 (2)
C5—H5	0.9500	C26—H26	0.9500
C6—C7	1.451 (2)	C27—C28	1.457 (2)
C7—C8	1.407 (2)	C28—C29	1.404 (2)
C8—C9	1.374 (2)	C29—C30	1.373 (2)
C9—C10	1.483 (2)	C30—C31	1.483 (2)
C10—H10A	0.9800	C31—H31A	0.9800
C10—H10B	0.9800	C31—H31B	0.9800
C10—H10C	0.9800	C31—H31C	0.9800
C11—H11A	0.9800	C32—H32A	0.9800
C11—H11B	0.9800	C32—H32B	0.9800
C11—H11C	0.9800	C32—H32C	0.9800
C12—C13	1.521 (2)	C33—C34	1.524 (2)
C12—H12A	0.9900	C33—H33A	0.9900
C12—H12B	0.9900	C33—H33B	0.9900
C14—C15	1.508 (2)	C35—C36	1.506 (3)
C14—H14A	0.9900	C35—H35A	0.9900
C14—H14B	0.9900	C35—H35B	0.9900
C15—C20	1.391 (2)	C36—C41	1.386 (3)
C15—C16	1.391 (2)	C36—C37	1.392 (3)
C16—C17	1.392 (2)	C37—C38	1.387 (3)
C16—H16	0.9500	C37—H37	0.9500
C17—C18	1.384 (2)	C38—C39	1.393 (3)
C18—C19	1.375 (3)	C39—C40	1.370 (4)
C18—H18	0.9500	C39—H39	0.9500
C19—C20	1.388 (3)	C40—C41	1.395 (3)
C19—H19	0.9500	C40—H40	0.9500
C20—H20	0.9500	C41—H41	0.9500
C21—H21A	0.9800	C42—H42A	0.9800
C21—H21B	0.9800	C42—H42B	0.9800
C21—H21C	0.9800	C42—H42C	0.9800
			
O2—S1—O1	118.99 (9)	O6—S2—O5	119.27 (9)
O2—S1—N1	107.99 (8)	O6—S2—N5	107.37 (8)
O1—S1—N1	107.54 (8)	O5—S2—N5	107.64 (8)
O2—S1—C1	106.52 (8)	O6—S2—C22	106.94 (8)
O1—S1—C1	109.78 (8)	O5—S2—C22	110.05 (9)
N1—S1—C1	105.20 (7)	N5—S2—C22	104.61 (8)
C17—O4—C21	118.06 (14)	C38—O8—C42	116.66 (15)
C8—N1—C11	117.85 (13)	C29—N5—C32	115.53 (14)
C8—N1—S1	112.72 (11)	C29—N5—S2	110.68 (11)
C11—N1—S1	119.23 (11)	C32—N5—S2	118.20 (12)
C7—N2—N3	103.82 (12)	C28—N6—N7	103.79 (12)
N2—N3—C9	114.16 (12)	C30—N7—N6	113.93 (12)
N2—N3—C12	118.52 (13)	C30—N7—C33	127.25 (13)
C9—N3—C12	127.16 (14)	N6—N7—C33	118.60 (12)
C13—N4—C14	121.04 (13)	C34—N8—C35	123.75 (14)
C13—N4—H4N	119.5	C34—N8—H8N	118.1
C14—N4—H4N	119.5	C35—N8—H8N	118.1
C2—C1—C6	121.61 (16)	C23—C22—C27	121.50 (17)
C2—C1—S1	119.22 (13)	C23—C22—S2	120.63 (15)
C6—C1—S1	119.08 (12)	C27—C22—S2	117.79 (12)
C3—C2—C1	119.14 (17)	C24—C23—C22	119.02 (18)
C3—C2—H2	120.4	C24—C23—H23	120.5
C1—C2—H2	120.4	C22—C23—H23	120.5
C2—C3—C4	120.21 (17)	C23—C24—C25	120.53 (17)
C2—C3—H3	119.9	C23—C24—H24	119.7
C4—C3—H3	119.9	C25—C24—H24	119.7
C5—C4—C3	120.80 (17)	C26—C25—C24	120.52 (19)
C5—C4—H4	119.6	C26—C25—H25	119.7
C3—C4—H4	119.6	C24—C25—H25	119.7
C4—C5—C6	120.19 (16)	C25—C26—C27	120.24 (18)
C4—C5—H5	119.9	C25—C26—H26	119.9
C6—C5—H5	119.9	C27—C26—H26	119.9
C5—C6—C1	117.99 (15)	C26—C27—C22	118.10 (15)
C5—C6—C7	123.80 (15)	C26—C27—C28	123.83 (15)
C1—C6—C7	117.92 (14)	C22—C27—C28	117.91 (15)
N2—C7—C8	110.69 (14)	N6—C28—C29	110.78 (14)
N2—C7—C6	125.17 (14)	N6—C28—C27	125.66 (14)
C8—C7—C6	123.98 (14)	C29—C28—C27	123.52 (14)
C9—C8—C7	106.65 (13)	C30—C29—C28	106.71 (14)
C9—C8—N1	128.31 (14)	C30—C29—N5	128.52 (15)
C7—C8—N1	124.90 (14)	C28—C29—N5	124.72 (14)
N3—C9—C8	104.64 (14)	N7—C30—C29	104.76 (13)
N3—C9—C10	123.84 (14)	N7—C30—C31	124.18 (15)
C8—C9—C10	131.52 (15)	C29—C30—C31	131.01 (15)
C9—C10—H10A	109.5	C30—C31—H31A	109.5
C9—C10—H10B	109.5	C30—C31—H31B	109.5
H10A—C10—H10B	109.5	H31A—C31—H31B	109.5
C9—C10—H10C	109.5	C30—C31—H31C	109.5
H10A—C10—H10C	109.5	H31A—C31—H31C	109.5
H10B—C10—H10C	109.5	H31B—C31—H31C	109.5
N1—C11—H11A	109.5	N5—C32—H32A	109.5
N1—C11—H11B	109.5	N5—C32—H32B	109.5
H11A—C11—H11B	109.5	H32A—C32—H32B	109.5
N1—C11—H11C	109.5	N5—C32—H32C	109.5
H11A—C11—H11C	109.5	H32A—C32—H32C	109.5
H11B—C11—H11C	109.5	H32B—C32—H32C	109.5
N3—C12—C13	111.23 (13)	N7—C33—C34	110.42 (13)
N3—C12—H12A	109.4	N7—C33—H33A	109.6
C13—C12—H12A	109.4	C34—C33—H33A	109.6
N3—C12—H12B	109.4	N7—C33—H33B	109.6
C13—C12—H12B	109.4	C34—C33—H33B	109.6
H12A—C12—H12B	108.0	H33A—C33—H33B	108.1
O3—C13—N4	124.14 (15)	O7—C34—N8	124.70 (15)
O3—C13—C12	121.12 (14)	O7—C34—C33	121.08 (14)
N4—C13—C12	114.73 (13)	N8—C34—C33	114.21 (13)
N4—C14—C15	114.83 (13)	N8—C35—C36	110.90 (14)
N4—C14—H14A	108.6	N8—C35—H35A	109.5
C15—C14—H14A	108.6	C36—C35—H35A	109.5
N4—C14—H14B	108.6	N8—C35—H35B	109.5
C15—C14—H14B	108.6	C36—C35—H35B	109.5
H14A—C14—H14B	107.5	H35A—C35—H35B	108.0
C20—C15—C16	119.23 (15)	C41—C36—C37	119.54 (19)
C20—C15—C14	117.34 (15)	C41—C36—C35	121.41 (19)
C16—C15—C14	123.39 (15)	C37—C36—C35	119.03 (17)
C15—C16—C17	119.81 (16)	C38—C37—C36	120.55 (17)
C15—C16—H16	120.1	C38—C37—H37	119.7
C17—C16—H16	120.1	C36—C37—H37	119.7
O4—C17—C18	115.50 (15)	O8—C38—C37	123.90 (18)
O4—C17—C16	123.79 (16)	O8—C38—C39	116.50 (18)
C18—C17—C16	120.71 (16)	C37—C38—C39	119.6 (2)
C19—C18—C17	119.30 (16)	C40—C39—C38	119.8 (2)
C19—C18—H18	120.4	C40—C39—H39	120.1
C17—C18—H18	120.4	C38—C39—H39	120.1
C18—C19—C20	120.76 (17)	C39—C40—C41	121.0 (2)
C18—C19—H19	119.6	C39—C40—H40	119.5
C20—C19—H19	119.6	C41—C40—H40	119.5
C19—C20—C15	120.16 (17)	C36—C41—C40	119.5 (2)
C19—C20—H20	119.9	C36—C41—H41	120.2
C15—C20—H20	119.9	C40—C41—H41	120.2
O4—C21—H21A	109.5	O8—C42—H42A	109.5
O4—C21—H21B	109.5	O8—C42—H42B	109.5
H21A—C21—H21B	109.5	H42A—C42—H42B	109.5
O4—C21—H21C	109.5	O8—C42—H42C	109.5
H21A—C21—H21C	109.5	H42A—C42—H42C	109.5
H21B—C21—H21C	109.5	H42B—C42—H42C	109.5
			
O2—S1—N1—C8	−70.02 (13)	O6—S2—N5—C29	−64.49 (13)
O1—S1—N1—C8	160.41 (12)	O5—S2—N5—C29	165.94 (12)
C1—S1—N1—C8	43.43 (13)	C22—S2—N5—C29	48.89 (13)
O2—S1—N1—C11	145.41 (13)	O6—S2—N5—C32	159.02 (14)
O1—S1—N1—C11	15.84 (15)	O5—S2—N5—C32	29.45 (16)
C1—S1—N1—C11	−101.13 (14)	C22—S2—N5—C32	−87.59 (14)
C7—N2—N3—C9	−1.96 (17)	C28—N6—N7—C30	−1.62 (17)
C7—N2—N3—C12	−177.68 (13)	C28—N6—N7—C33	−176.66 (13)
O2—S1—C1—C2	−99.39 (15)	O6—S2—C22—C23	−103.66 (15)
O1—S1—C1—C2	30.69 (17)	O5—S2—C22—C23	27.27 (18)
N1—S1—C1—C2	146.13 (14)	N5—S2—C22—C23	142.65 (15)
O2—S1—C1—C6	77.32 (14)	O6—S2—C22—C27	73.01 (15)
O1—S1—C1—C6	−152.60 (13)	O5—S2—C22—C27	−156.06 (13)
N1—S1—C1—C6	−37.16 (15)	N5—S2—C22—C27	−40.69 (15)
C6—C1—C2—C3	−1.9 (3)	C27—C22—C23—C24	−2.3 (3)
S1—C1—C2—C3	174.73 (15)	S2—C22—C23—C24	174.25 (15)
C1—C2—C3—C4	−0.4 (3)	C22—C23—C24—C25	−0.4 (3)
C2—C3—C4—C5	1.7 (3)	C23—C24—C25—C26	2.1 (3)
C3—C4—C5—C6	−0.7 (3)	C24—C25—C26—C27	−1.1 (3)
C4—C5—C6—C1	−1.5 (2)	C25—C26—C27—C22	−1.5 (2)
C4—C5—C6—C7	172.30 (16)	C25—C26—C27—C28	173.67 (16)
C2—C1—C6—C5	2.8 (2)	C23—C22—C27—C26	3.2 (2)
S1—C1—C6—C5	−173.81 (12)	S2—C22—C27—C26	−173.39 (12)
C2—C1—C6—C7	−171.34 (15)	C23—C22—C27—C28	−172.24 (16)
S1—C1—C6—C7	12.0 (2)	S2—C22—C27—C28	11.1 (2)
N3—N2—C7—C8	0.75 (17)	N7—N6—C28—C29	1.21 (17)
N3—N2—C7—C6	−174.70 (14)	N7—N6—C28—C27	−176.35 (15)
C5—C6—C7—N2	10.3 (2)	C26—C27—C28—N6	14.7 (3)
C1—C6—C7—N2	−175.85 (15)	C22—C27—C28—N6	−170.05 (15)
C5—C6—C7—C8	−164.52 (16)	C26—C27—C28—C29	−162.53 (16)
C1—C6—C7—C8	9.3 (2)	C22—C27—C28—C29	12.7 (2)
N2—C7—C8—C9	0.62 (18)	N6—C28—C29—C30	−0.46 (18)
C6—C7—C8—C9	176.14 (14)	C27—C28—C29—C30	177.17 (15)
N2—C7—C8—N1	−175.25 (15)	N6—C28—C29—N5	−178.22 (15)
C6—C7—C8—N1	0.3 (3)	C27—C28—C29—N5	−0.6 (3)
C11—N1—C8—C9	−60.2 (2)	C32—N5—C29—C30	−73.5 (2)
S1—N1—C8—C9	154.68 (14)	S2—N5—C29—C30	148.71 (15)
C11—N1—C8—C7	114.74 (18)	C32—N5—C29—C28	103.7 (2)
S1—N1—C8—C7	−30.4 (2)	S2—N5—C29—C28	−34.0 (2)
N2—N3—C9—C8	2.36 (18)	N6—N7—C30—C29	1.36 (18)
C12—N3—C9—C8	177.63 (14)	C33—N7—C30—C29	175.89 (14)
N2—N3—C9—C10	−177.77 (15)	N6—N7—C30—C31	179.17 (15)
C12—N3—C9—C10	−2.5 (3)	C33—N7—C30—C31	−6.3 (3)
C7—C8—C9—N3	−1.71 (17)	C28—C29—C30—N7	−0.52 (17)
N1—C8—C9—N3	173.98 (15)	N5—C29—C30—N7	177.14 (16)
C7—C8—C9—C10	178.43 (17)	C28—C29—C30—C31	−178.12 (17)
N1—C8—C9—C10	−5.9 (3)	N5—C29—C30—C31	−0.5 (3)
N2—N3—C12—C13	82.23 (17)	C30—N7—C33—C34	−101.77 (18)
C9—N3—C12—C13	−92.87 (19)	N6—N7—C33—C34	72.54 (17)
C14—N4—C13—O3	3.8 (2)	C35—N8—C34—O7	−1.8 (3)
C14—N4—C13—C12	−175.58 (14)	C35—N8—C34—C33	179.52 (15)
N3—C12—C13—O3	26.5 (2)	N7—C33—C34—O7	44.81 (19)
N3—C12—C13—N4	−154.08 (14)	N7—C33—C34—N8	−136.44 (14)
C13—N4—C14—C15	−171.18 (14)	C34—N8—C35—C36	−132.81 (17)
N4—C14—C15—C20	169.04 (15)	N8—C35—C36—C41	119.73 (19)
N4—C14—C15—C16	−13.3 (2)	N8—C35—C36—C37	−61.8 (2)
C20—C15—C16—C17	−1.1 (3)	C41—C36—C37—C38	0.2 (3)
C14—C15—C16—C17	−178.74 (16)	C35—C36—C37—C38	−178.29 (16)
C21—O4—C17—C18	169.25 (18)	C42—O8—C38—C37	7.5 (3)
C21—O4—C17—C16	−10.9 (3)	C42—O8—C38—C39	−173.53 (18)
C15—C16—C17—O4	−177.66 (16)	C36—C37—C38—O8	−179.58 (16)
C15—C16—C17—C18	2.1 (3)	C36—C37—C38—C39	1.5 (3)
O4—C17—C18—C19	178.50 (17)	O8—C38—C39—C40	179.12 (18)
C16—C17—C18—C19	−1.3 (3)	C37—C38—C39—C40	−1.9 (3)
C17—C18—C19—C20	−0.5 (3)	C38—C39—C40—C41	0.6 (3)
C18—C19—C20—C15	1.5 (3)	C37—C36—C41—C40	−1.4 (3)
C16—C15—C20—C19	−0.7 (3)	C35—C36—C41—C40	176.98 (18)
C14—C15—C20—C19	177.06 (16)	C39—C40—C41—C36	1.1 (3)

### Hydrogen-bond geometry (Å, º)

**Table:**

*D*—H···*A*	*D*—H	H···*A*	*D*···*A*	*D*—H···*A*
N4—H4*N*···O7^i^	0.88	2.12	2.9511 (17)	158
N8—H8*N*···O3^ii^	0.88	2.02	2.8928 (18)	171
C10—H10*C*···O6^iii^	0.98	2.45	3.349 (2)	152
C12—H12*A*···O7^i^	0.99	2.56	3.2099 (19)	123
C14—H14*A*···N6^ii^	0.99	2.58	3.556 (2)	168
C16—H16···O7^i^	0.95	2.43	3.369 (2)	172
C25—H25···O4^iv^	0.95	2.47	3.365 (2)	156
C31—H31*C*···O2^v^	0.98	2.49	3.203 (2)	129
C33—H33*A*···O3^ii^	0.99	2.46	3.320 (2)	145
C33—H33*B*···O5^vi^	0.99	2.45	3.352 (2)	151
C40—H40···O2	0.95	2.59	3.367 (3)	139

Symmetry codes: (i) −*x*+2, −*y*+1, −*z*+1; (ii) −*x*+1, −*y*+1, −*z*+1; (iii) −*x*+2, *y*+1/2, −*z*+3/2; (iv) *x*−1, *y*, *z*+1; (v) −*x*+1, *y*−1/2, −*z*+3/2; (vi) *x*, −*y*+1/2, *z*−1/2.

## References

[bb1] Ahmad, M., Siddiqui, H. L., Ahmad, S., Parvez, M. & Tizzard, G. J. (2010*a*). *J. Chem. Crystallogr.* **40**, 1188–1194.

[bb2] Ahmad, M., Siddiqui, H. L., Gardiner, J. M., Parvez, M. & Aslam, S. (2012). *Med. Chem. Res.* doi:10.1007/s00044-012-0062-6.

[bb3] Ahmad, M., Siddiqui, H. L., Khattak, M. I., Ahmad, S. & Parvez, M. (2011*a*). *Acta Cryst.* E**67**, o216–o217. 10.1107/S1600536810052177 PMC3050140 21522716

[bb4] Ahmad, M., Siddiqui, H. L., Khattak, M. I., Ahmad, S. & Parvez, M. (2011*b*). *Acta Cryst.* E**67**, o218–o219. 10.1107/S160053681005227X PMC3050389 21522717

[bb5] Ahmad, M., Siddiqui, H. L., Zia-ur-Rehman, M. & Parvez, M. (2010*b*). *Eur. J. Med. Chem.* **45**, 698–704. 10.1016/j.ejmech.2009.11.016 19962218

[bb6] Bruker (2004). *APEX2*, *SAINT* and *SADABS* Bruker AXS Inc., Madison, Wisconsin, USA.

[bb7] Farrugia, L. J. (1997). *J. Appl. Cryst.* **30**, 565.

[bb8] Lombardino, J. G., Wiseman, E. H. & Chiaini, J. (1973). *J. Med. Chem.* **16**, 493–496. 10.1021/jm00263a017 4198028

[bb9] Sheldrick, G. M. (2008). *Acta Cryst.* A**64**, 112–122. 10.1107/S0108767307043930 18156677

[bb10] Silverstein, F. E., Faich, G., Goldstein, J. L., Simon, L. S., Pincus, T., Whelton, A., Makuch, R., Eisen, G., Agrawal, N. M., Stenson, W. F., Burr, A. M., Zhao, W. W., Kent, J. D., Lefkowith, J. B., Verburg, K. M. & Geis, G. S. (2000). *J. Am. Med. Assoc.* **284**, 1247–1255. 10.1001/jama.284.10.1247 10979111

[bb11] Turck, D., Roth, W. & Busch, U. (1996). *Br. J. Rheumatol.* **35**, 13–16. 10.1093/rheumatology/35.suppl_1.13 8630630

[bb12] Zinnes, H., Lindo, N. A., Sircar, J. C., Schwartz, M. L. & Shavel, J. Jr (1973). *J. Med. Chem.* **16**, 44–48. 10.1021/jm00259a013 4682200

